# Supplementary figures and images for: Vitamin D Modified DSS-Induced Colitis in Mice via STING Signaling Pathway
Source: Biology (Basel). 2025 Jun 18;14(6):715. doi: 10.3390/biology14060715 (PMC12190092; doi:10.3390/biology14060715)

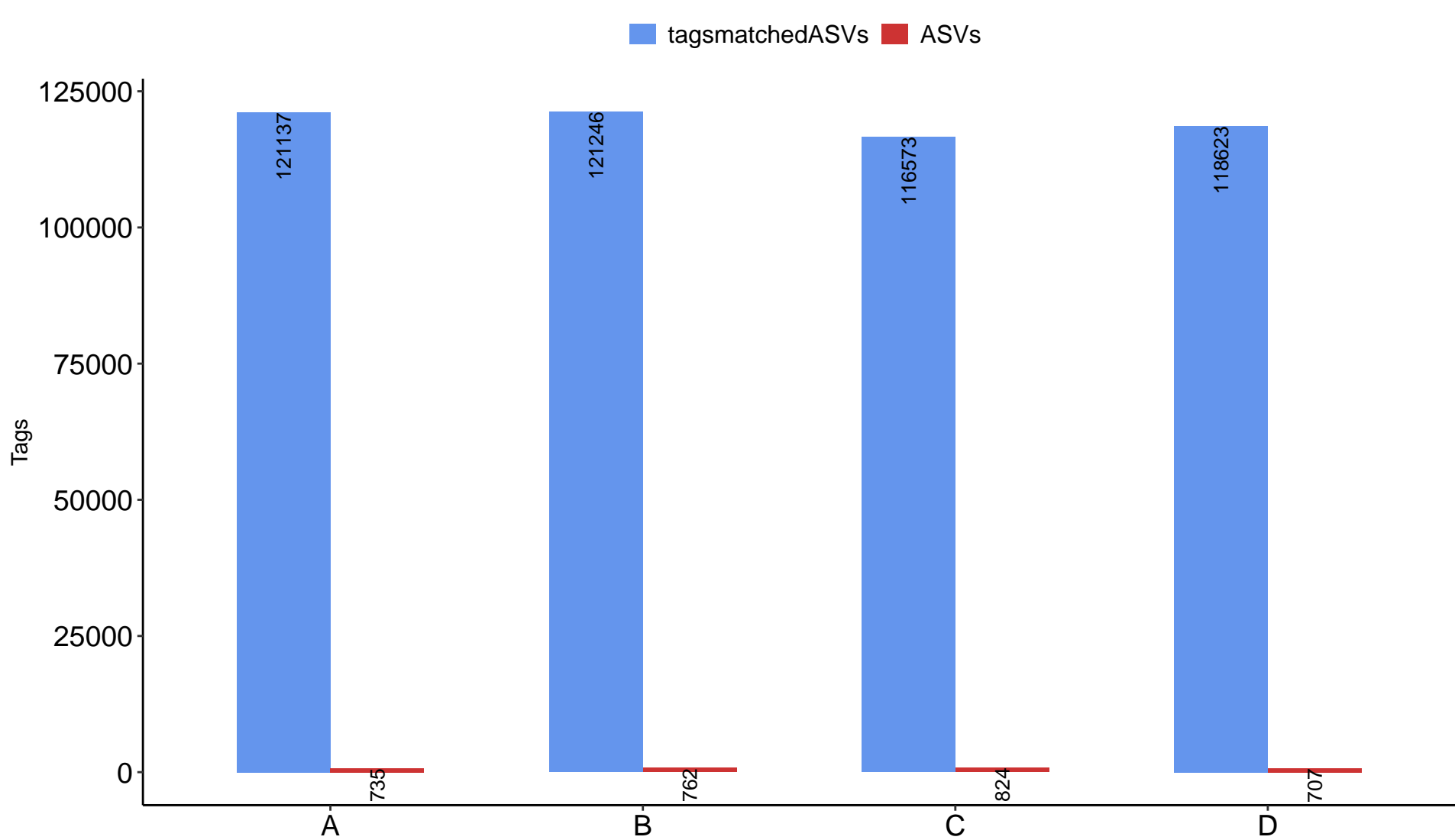

Supplement: Supplementary file 1 [file biology-14-00715-s001.zip › Supplementary Materials S1: 16s-report of gut microbiota/02_sequence_statistic/Group_Sample_stats_OTU.pdf]

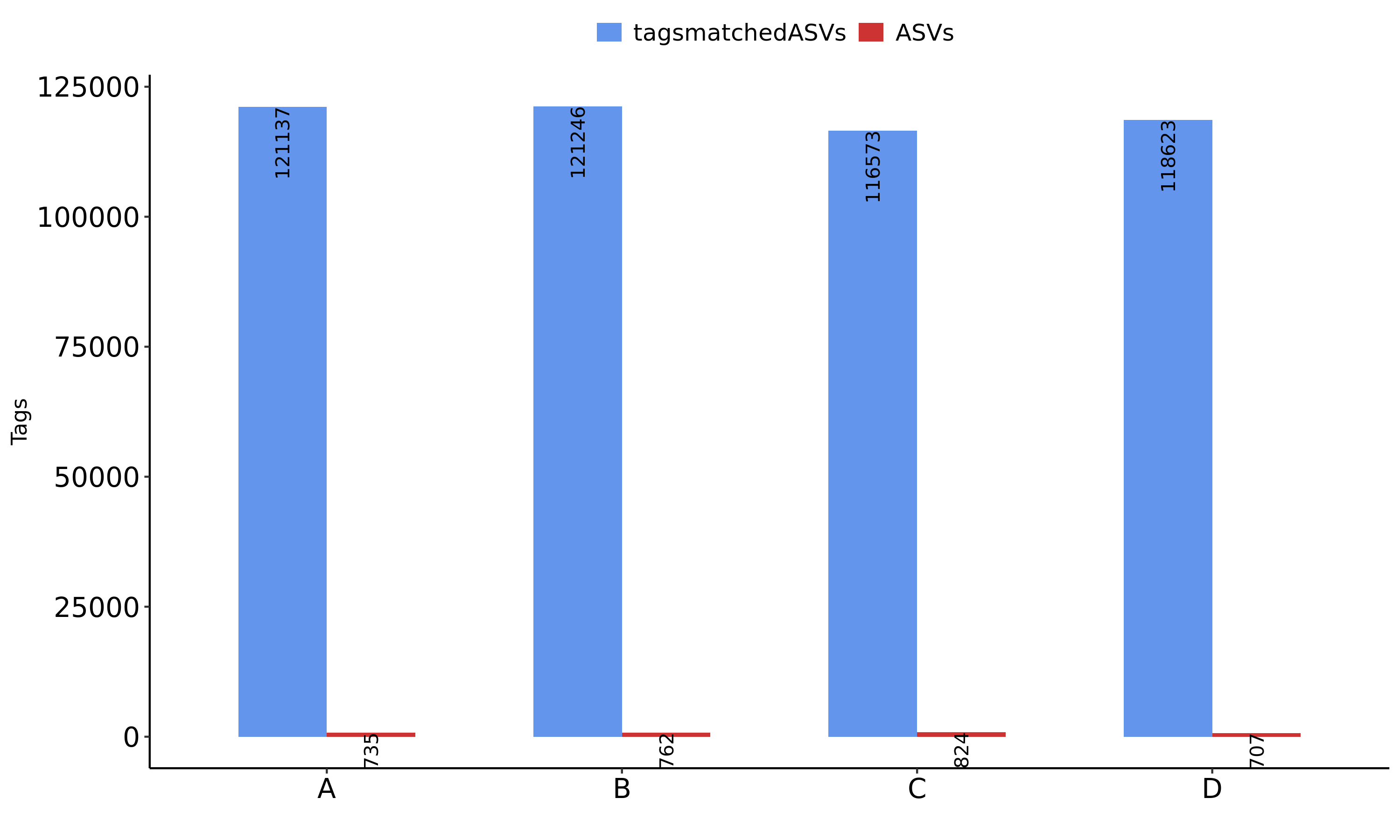

Supplement: Supplementary file 1 [file biology-14-00715-s001.zip › Supplementary Materials S1: 16s-report of gut microbiota/02_sequence_statistic/Group_Sample_stats_OTU.png]

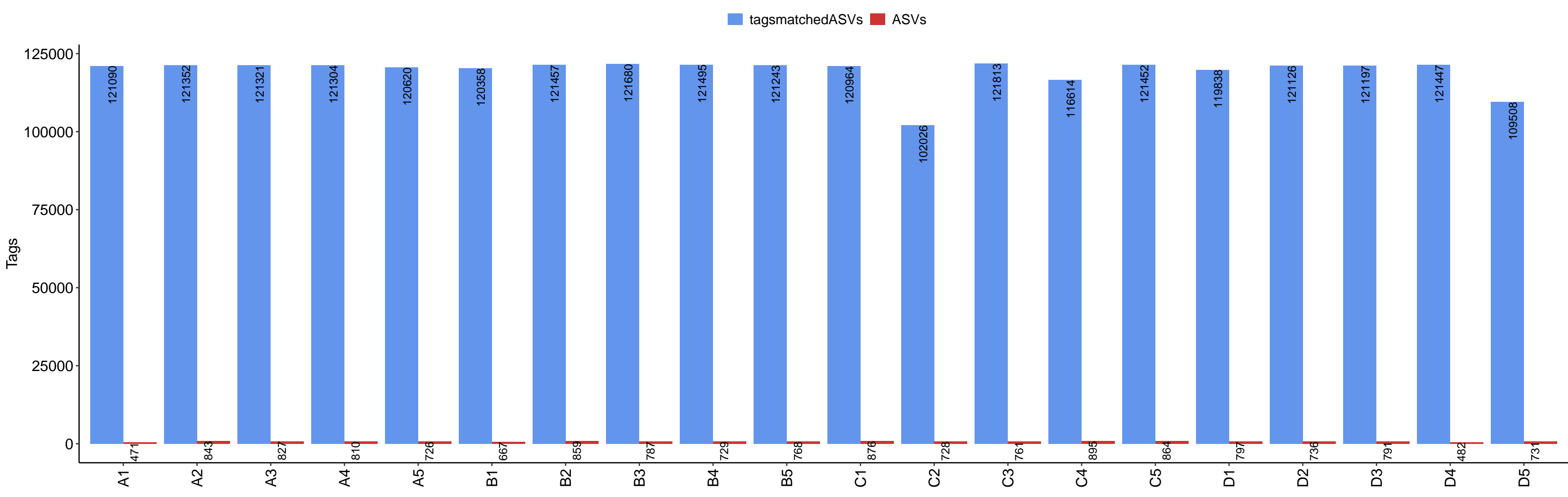

Supplement: Supplementary file 1 [file biology-14-00715-s001.zip › Supplementary Materials S1: 16s-report of gut microbiota/02_sequence_statistic/Sample_stats_OTU.pdf]

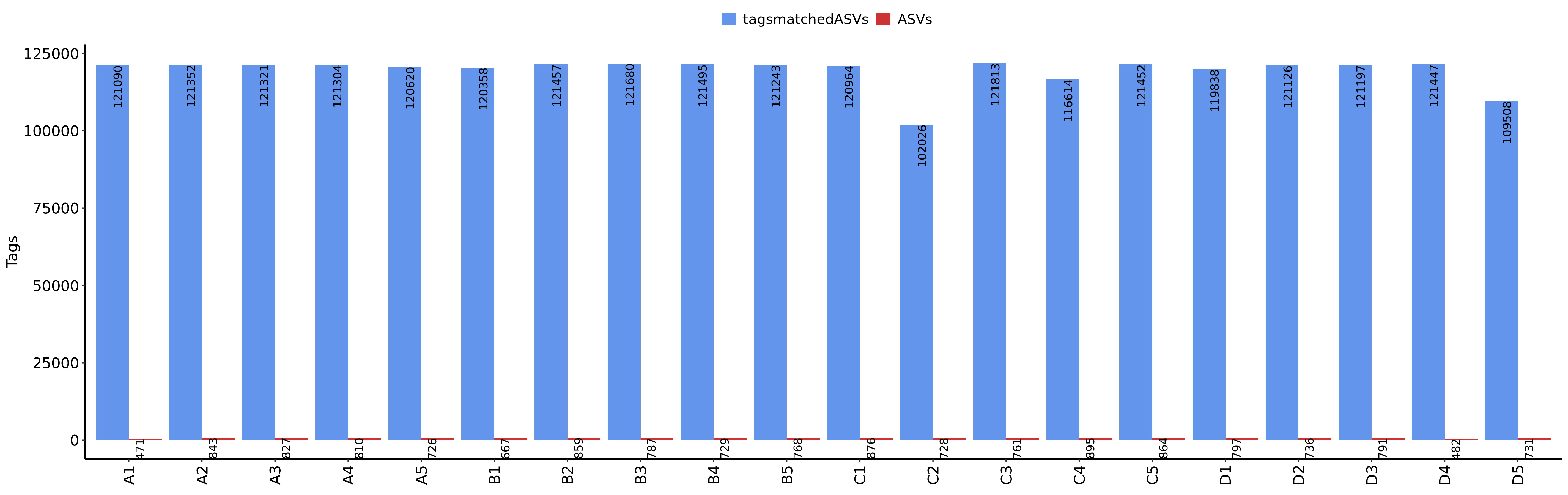

Supplement: Supplementary file 1 [file biology-14-00715-s001.zip › Supplementary Materials S1: 16s-report of gut microbiota/02_sequence_statistic/Sample_stats_OTU.png]

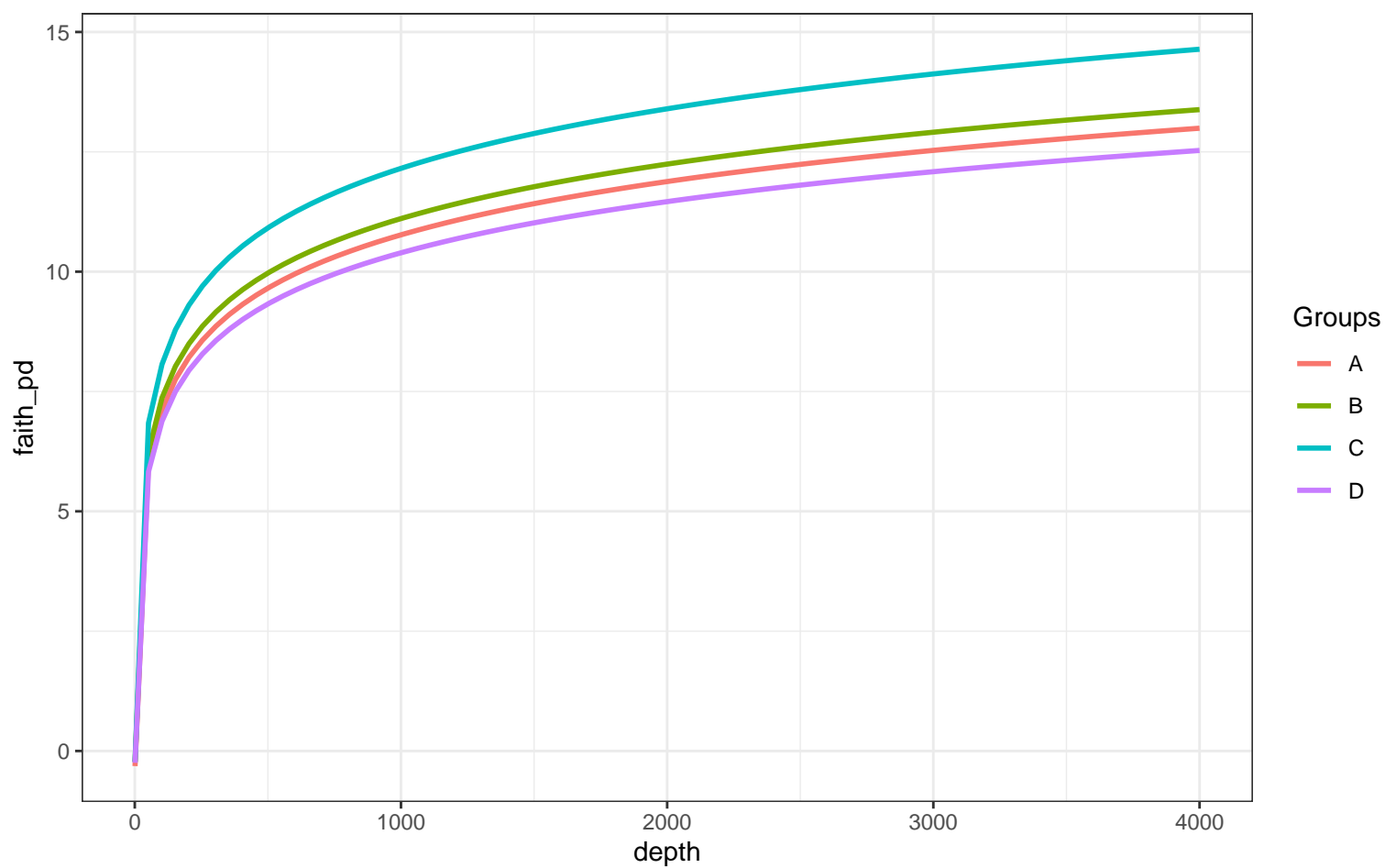

Supplement: Supplementary file 1 [file biology-14-00715-s001.zip › Supplementary Materials S1: 16s-report of gut microbiota/03_diversity-metrics/alpha_rarefaction/faith_pd_groups.pdf]

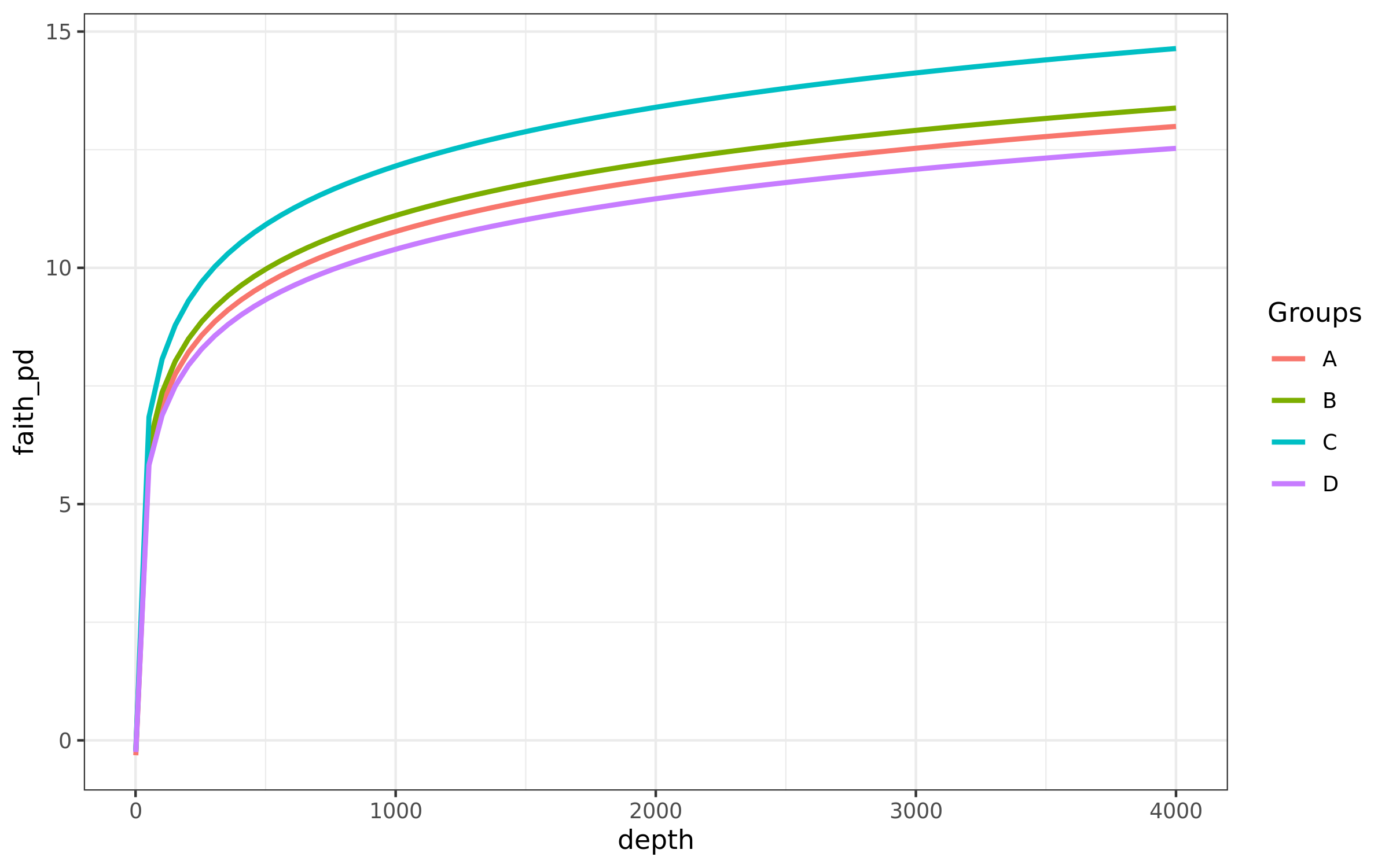

Supplement: Supplementary file 1 [file biology-14-00715-s001.zip › Supplementary Materials S1: 16s-report of gut microbiota/03_diversity-metrics/alpha_rarefaction/faith_pd_groups.png]

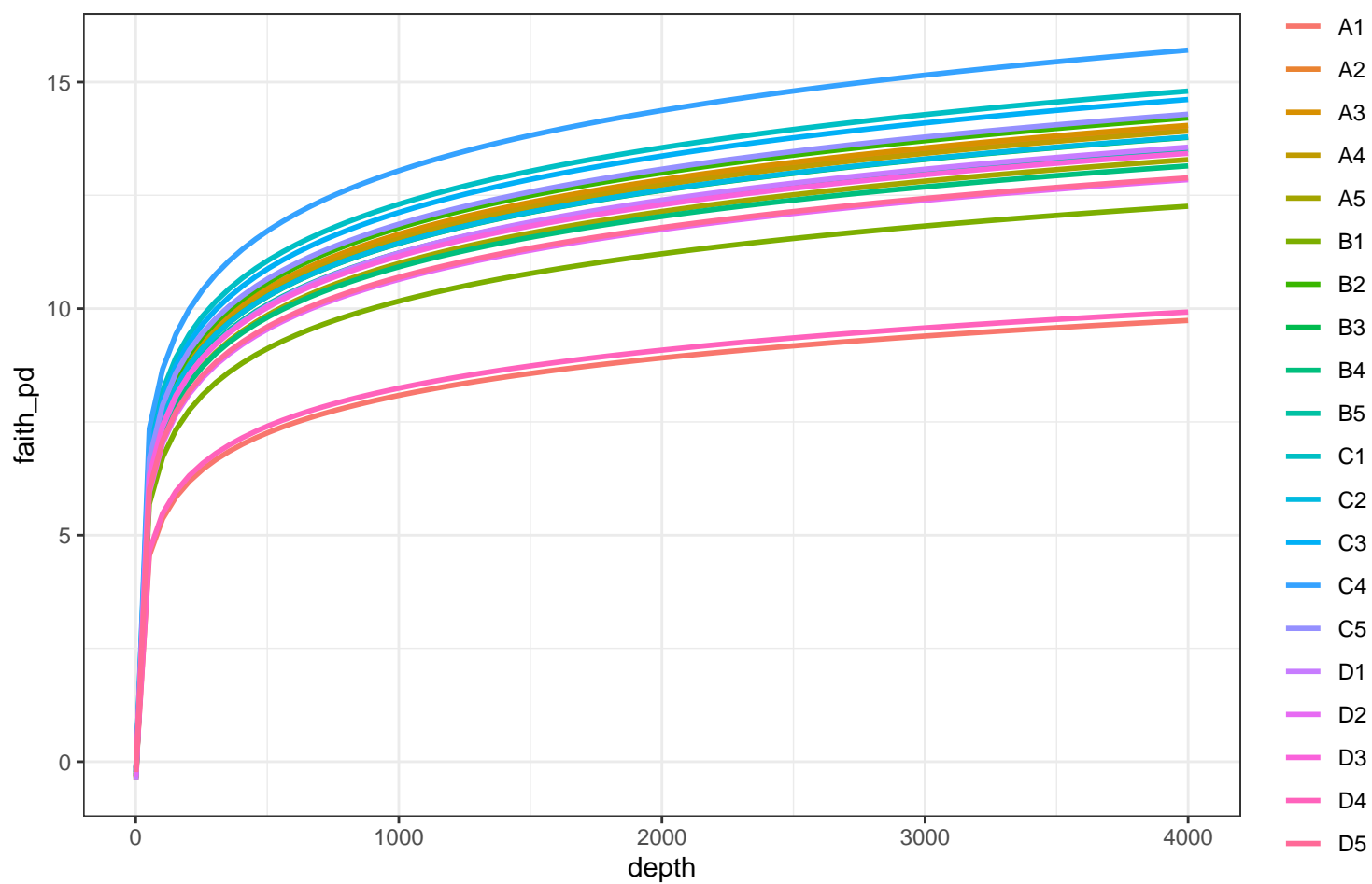

Supplement: Supplementary file 1 [file biology-14-00715-s001.zip › Supplementary Materials S1: 16s-report of gut microbiota/03_diversity-metrics/alpha_rarefaction/faith_pd_samples.pdf]

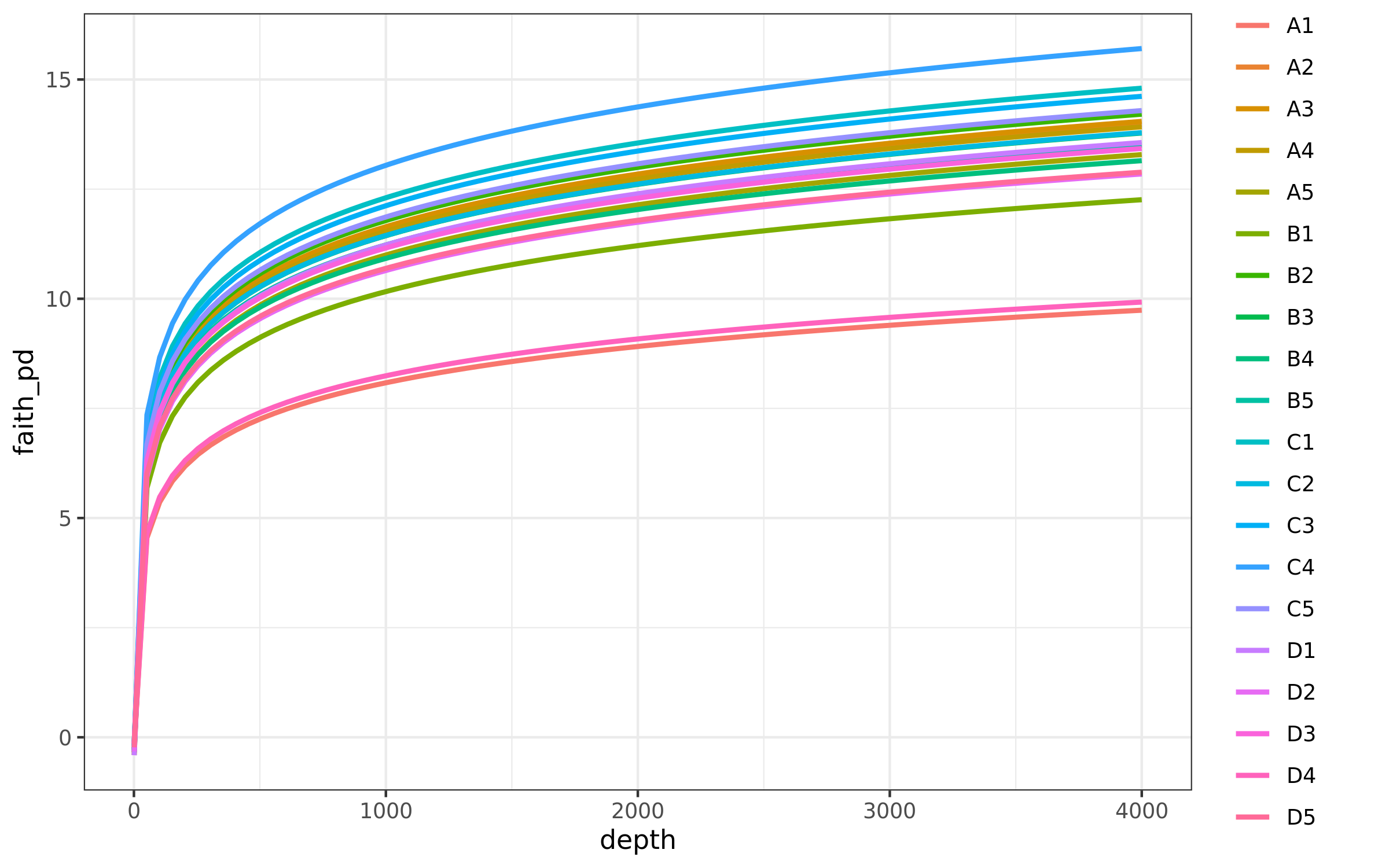

Supplement: Supplementary file 1 [file biology-14-00715-s001.zip › Supplementary Materials S1: 16s-report of gut microbiota/03_diversity-metrics/alpha_rarefaction/faith_pd_samples.png]

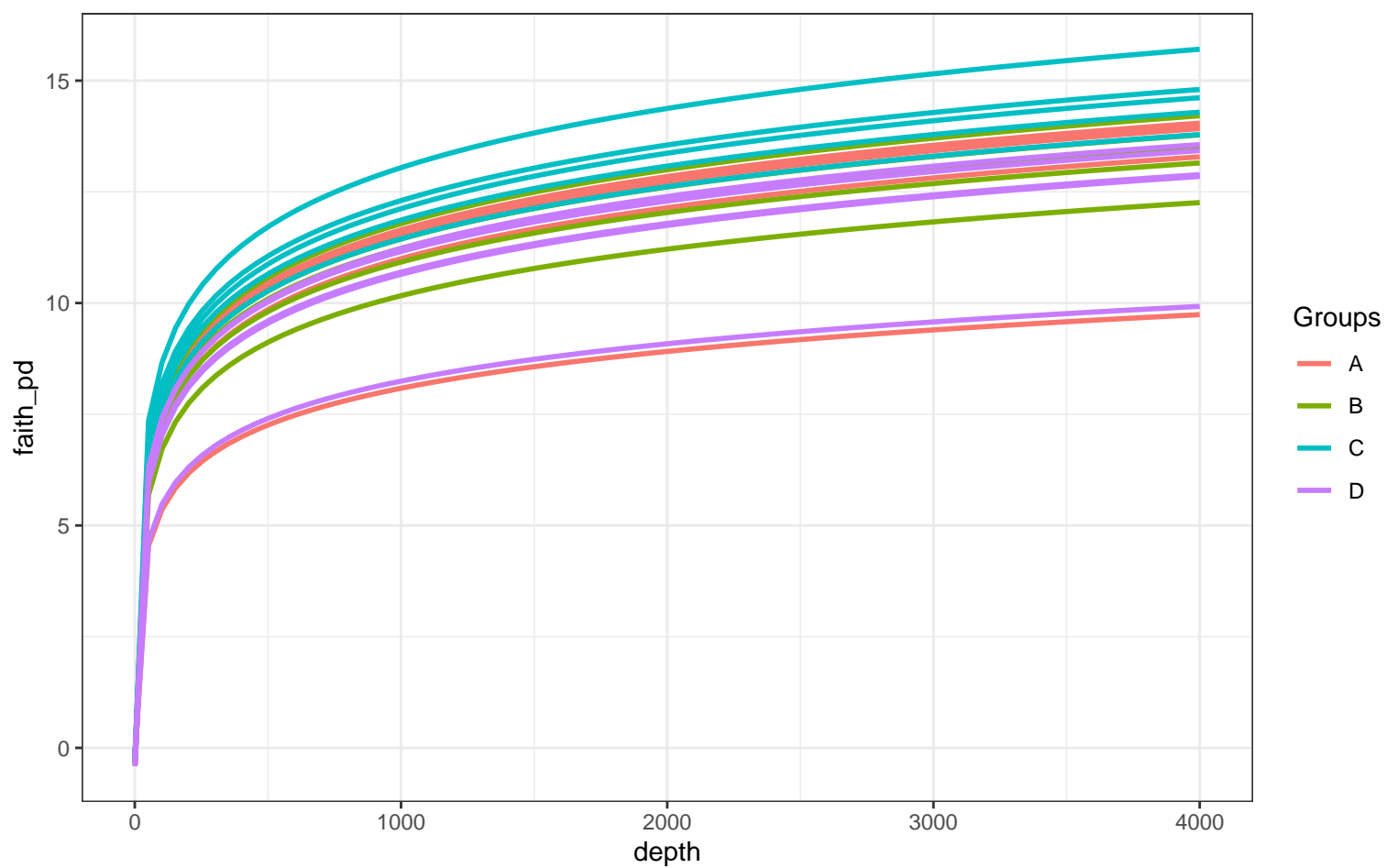

Supplement: Supplementary file 1 [file biology-14-00715-s001.zip › Supplementary Materials S1: 16s-report of gut microbiota/03_diversity-metrics/alpha_rarefaction/faith_pd_sample_GroupColor.pdf]

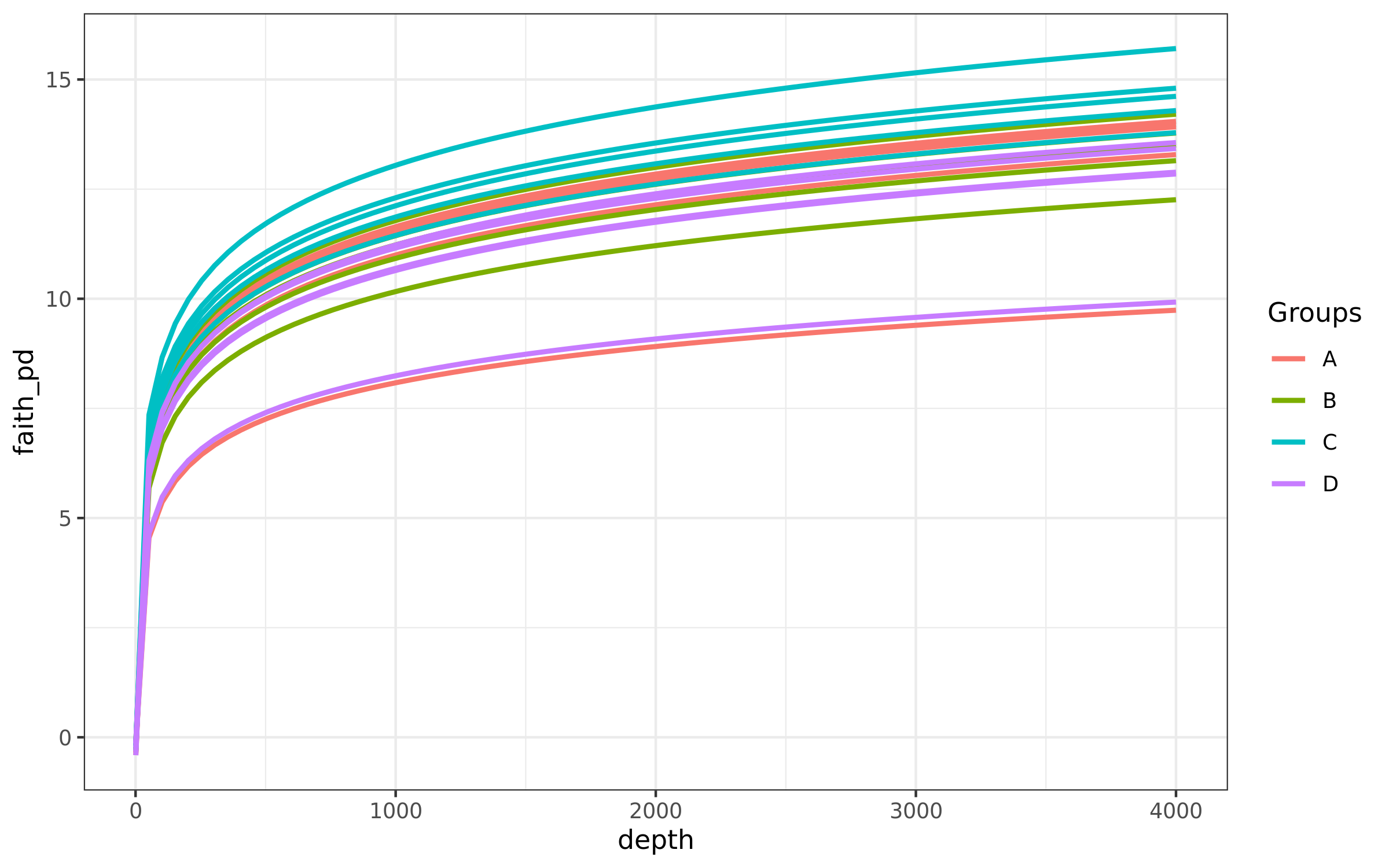

Supplement: Supplementary file 1 [file biology-14-00715-s001.zip › Supplementary Materials S1: 16s-report of gut microbiota/03_diversity-metrics/alpha_rarefaction/faith_pd_sample_GroupColor.png]

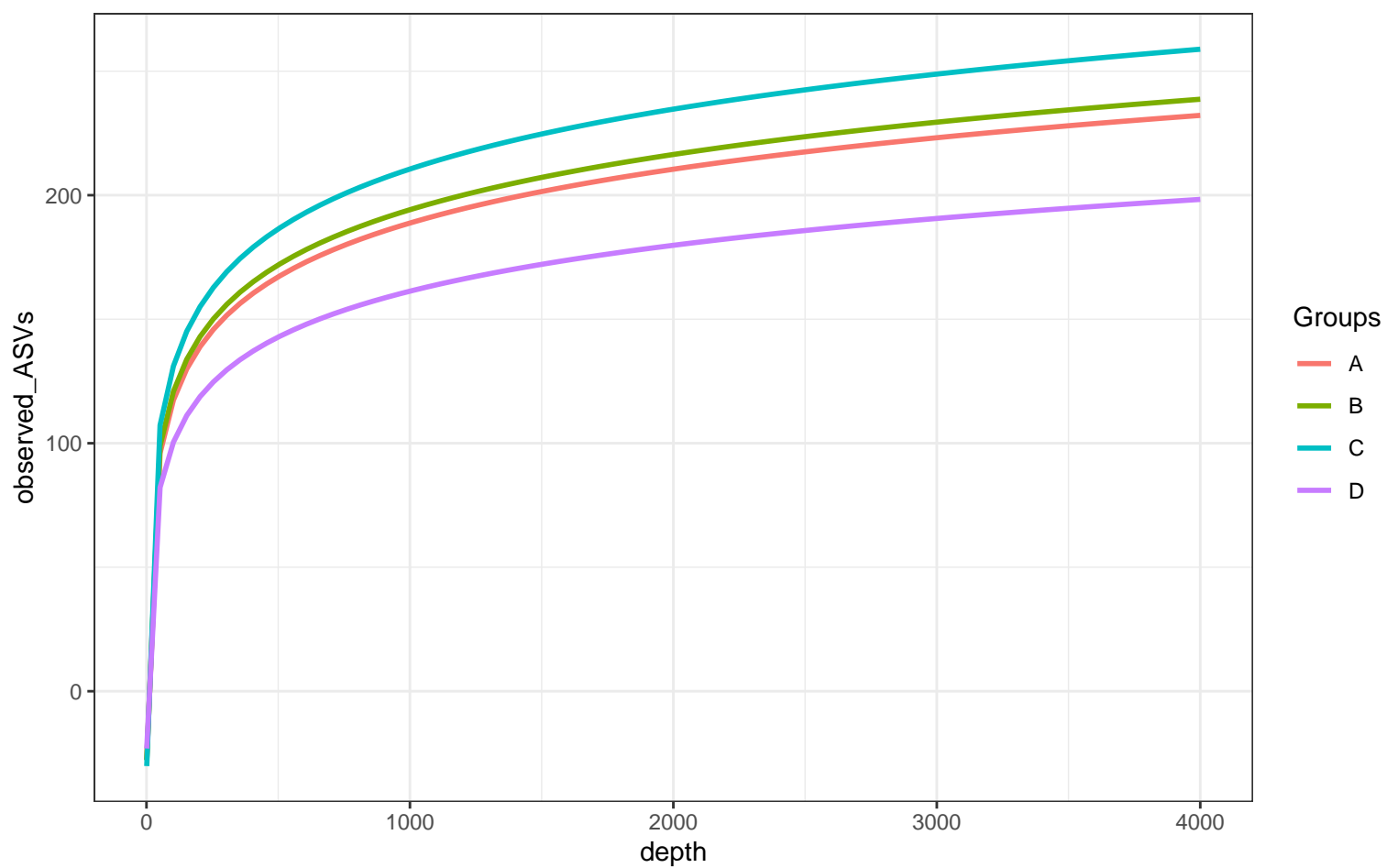

Supplement: Supplementary file 1 [file biology-14-00715-s001.zip › Supplementary Materials S1: 16s-report of gut microbiota/03_diversity-metrics/alpha_rarefaction/observed_otus_rarefaction_groups.pdf]

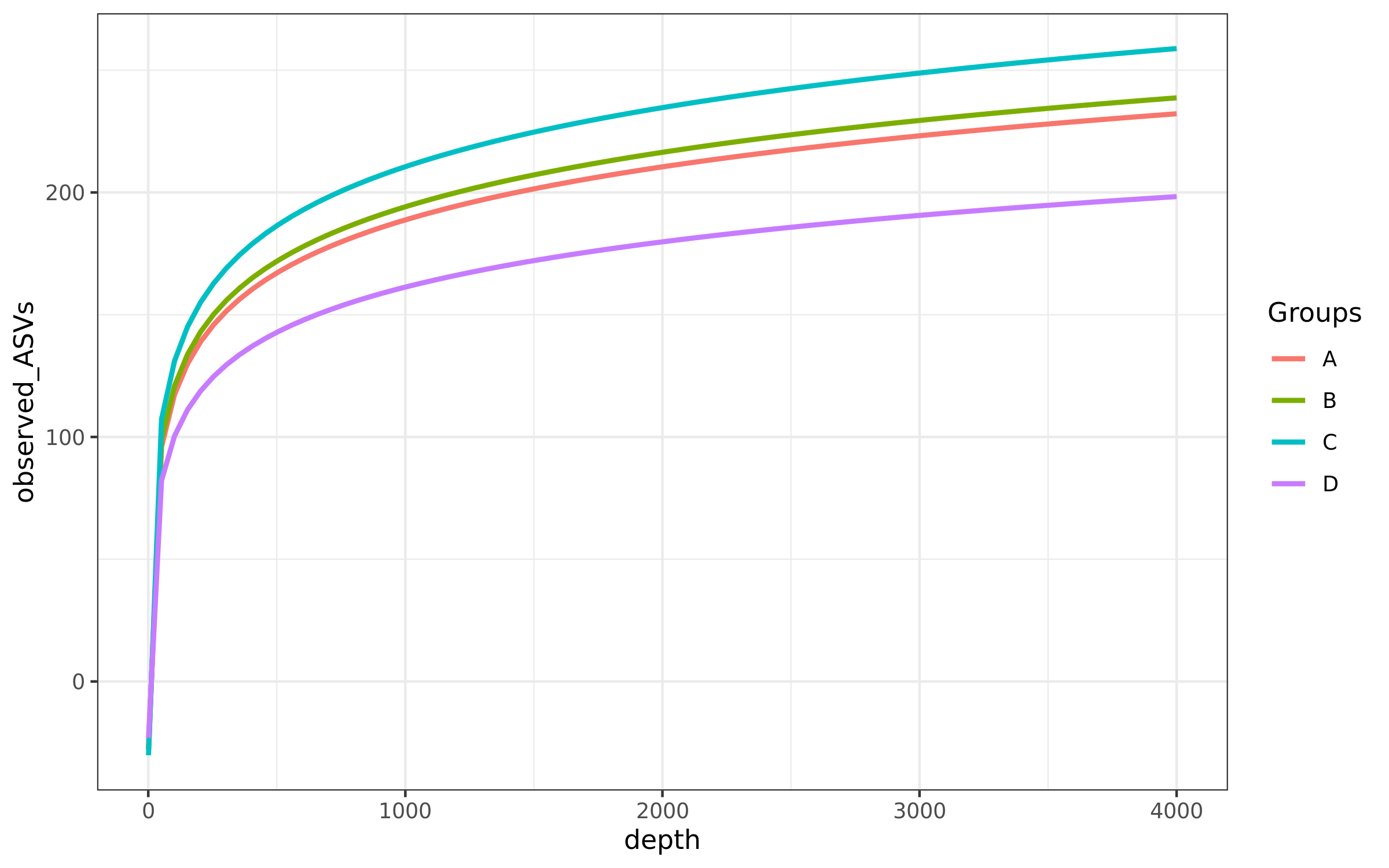

Supplement: Supplementary file 1 [file biology-14-00715-s001.zip › Supplementary Materials S1: 16s-report of gut microbiota/03_diversity-metrics/alpha_rarefaction/observed_otus_rarefaction_groups.png]

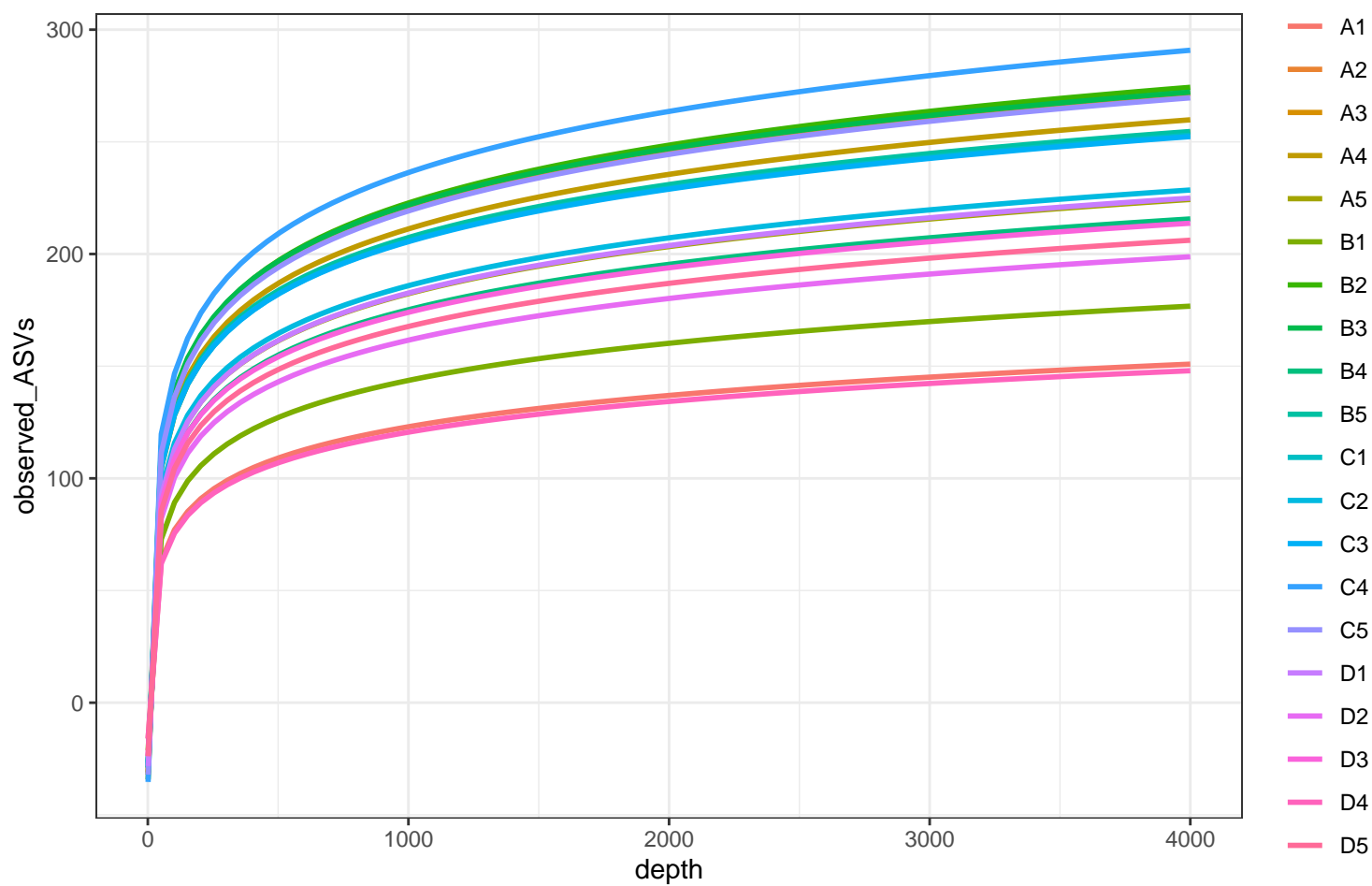

Supplement: Supplementary file 1 [file biology-14-00715-s001.zip › Supplementary Materials S1: 16s-report of gut microbiota/03_diversity-metrics/alpha_rarefaction/observed_otus_rarefaction_samples.pdf]

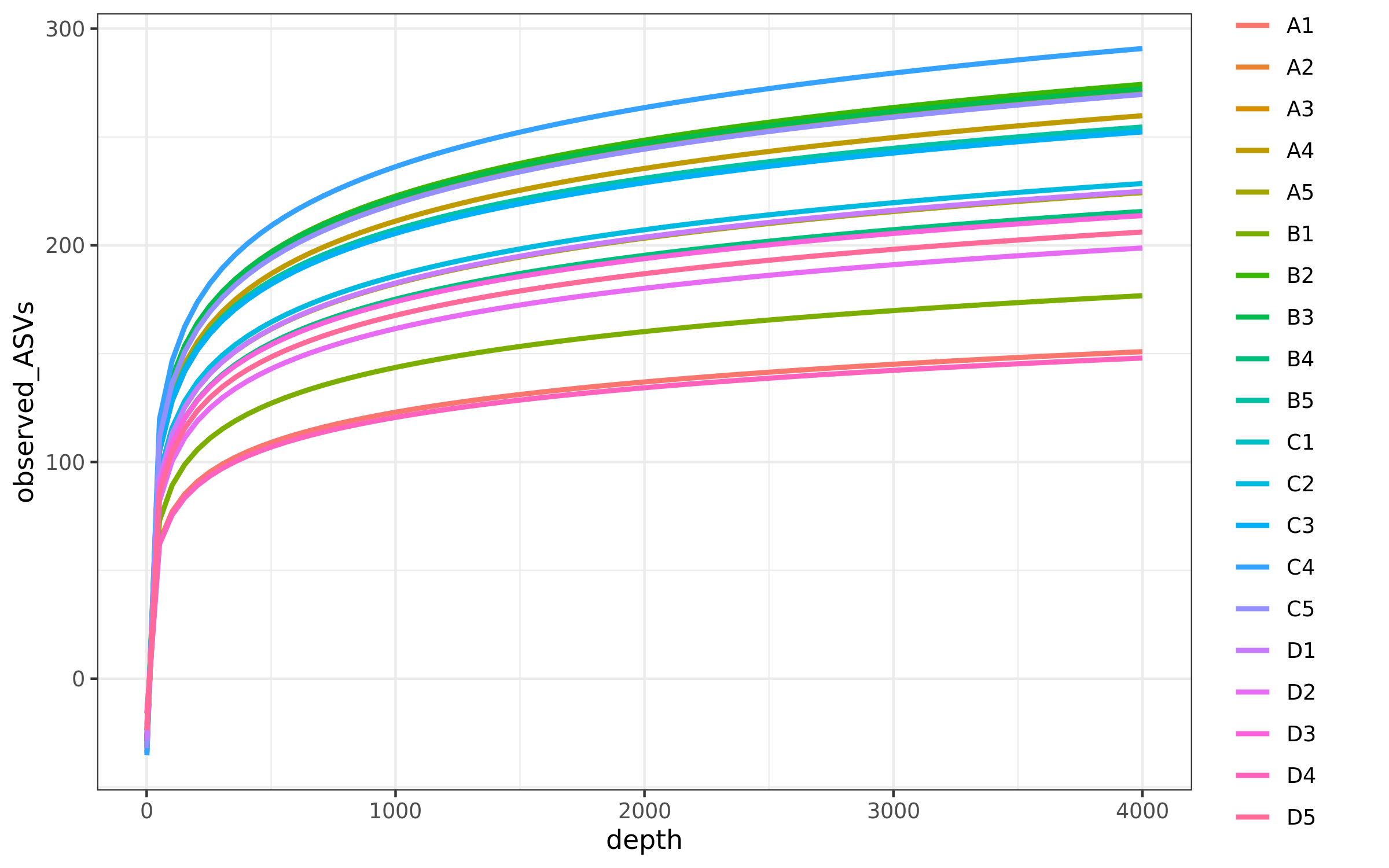

Supplement: Supplementary file 1 [file biology-14-00715-s001.zip › Supplementary Materials S1: 16s-report of gut microbiota/03_diversity-metrics/alpha_rarefaction/observed_otus_rarefaction_samples.png]

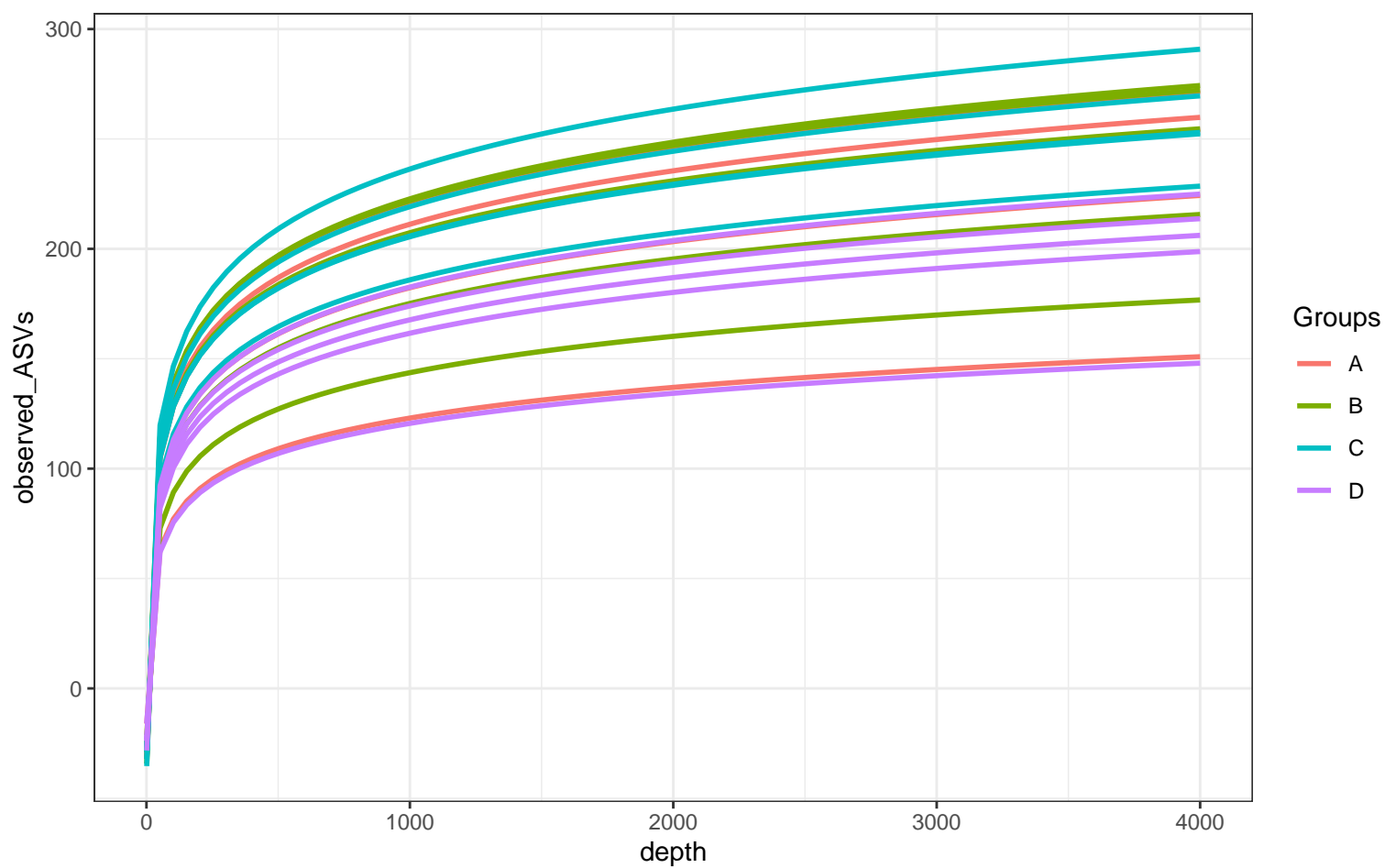

Supplement: Supplementary file 1 [file biology-14-00715-s001.zip › Supplementary Materials S1: 16s-report of gut microbiota/03_diversity-metrics/alpha_rarefaction/observed_otus_rarefaction_sample_GroupColor.pdf]

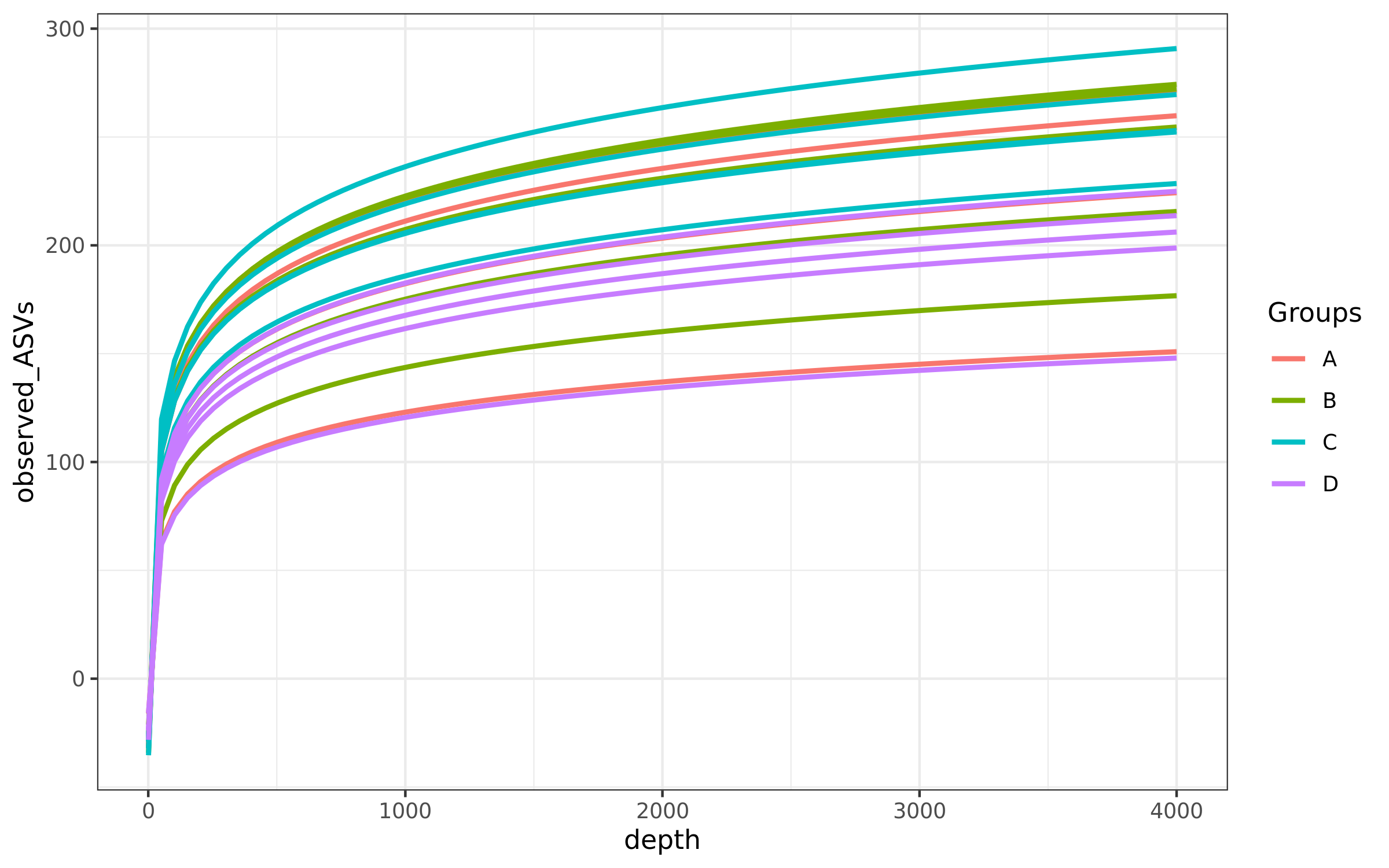

Supplement: Supplementary file 1 [file biology-14-00715-s001.zip › Supplementary Materials S1: 16s-report of gut microbiota/03_diversity-metrics/alpha_rarefaction/observed_otus_rarefaction_sample_GroupColor.png]

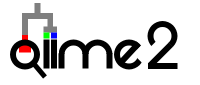

Supplement: Supplementary file 1 [file biology-14-00715-s001.zip › Supplementary Materials S1: 16s-report of gut microbiota/03_diversity-metrics/alpha_rarefaction/q2templateassets/img/qiime2-rect-200.png]

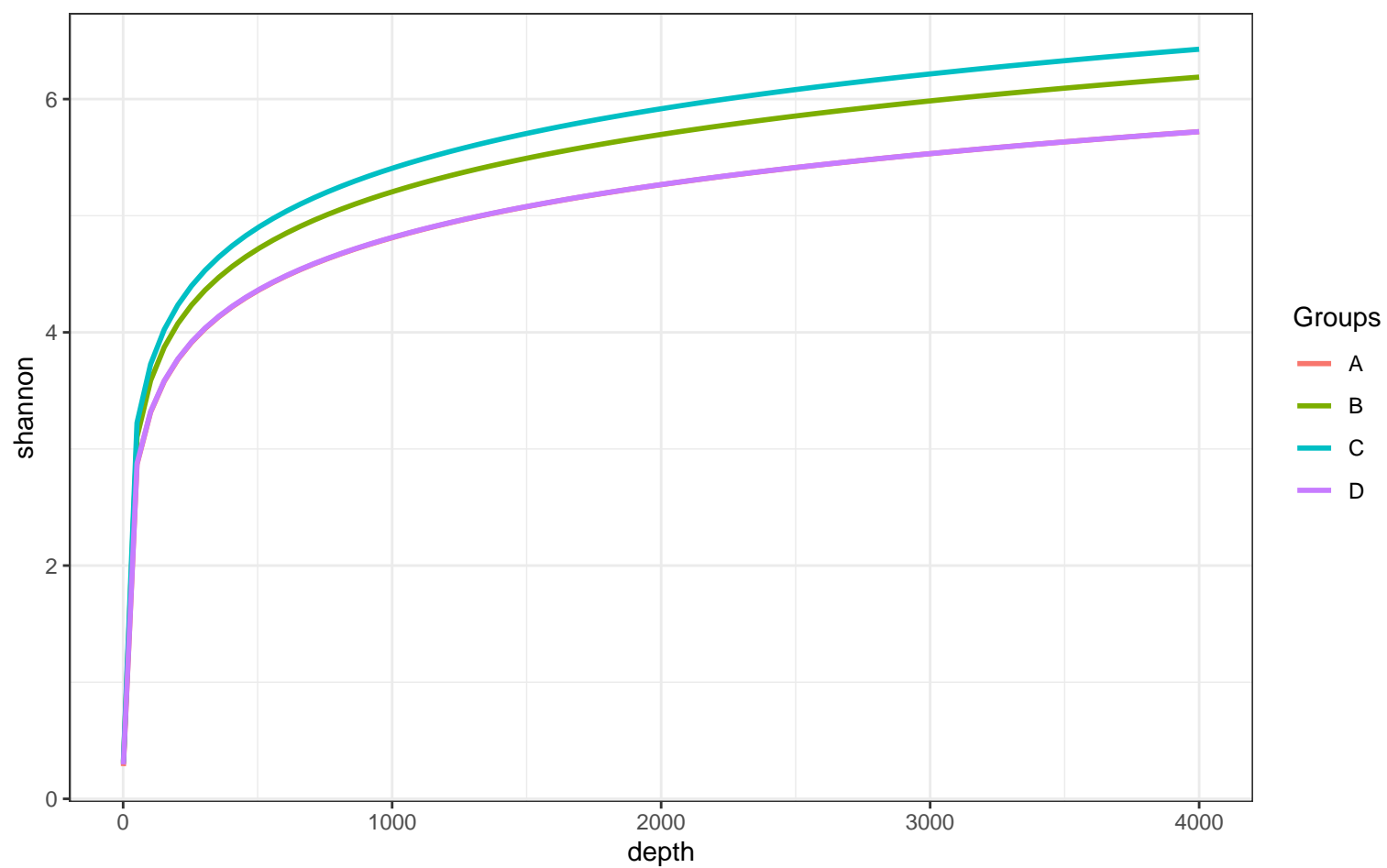

Supplement: Supplementary file 1 [file biology-14-00715-s001.zip › Supplementary Materials S1: 16s-report of gut microbiota/03_diversity-metrics/alpha_rarefaction/shannon_groups.pdf]

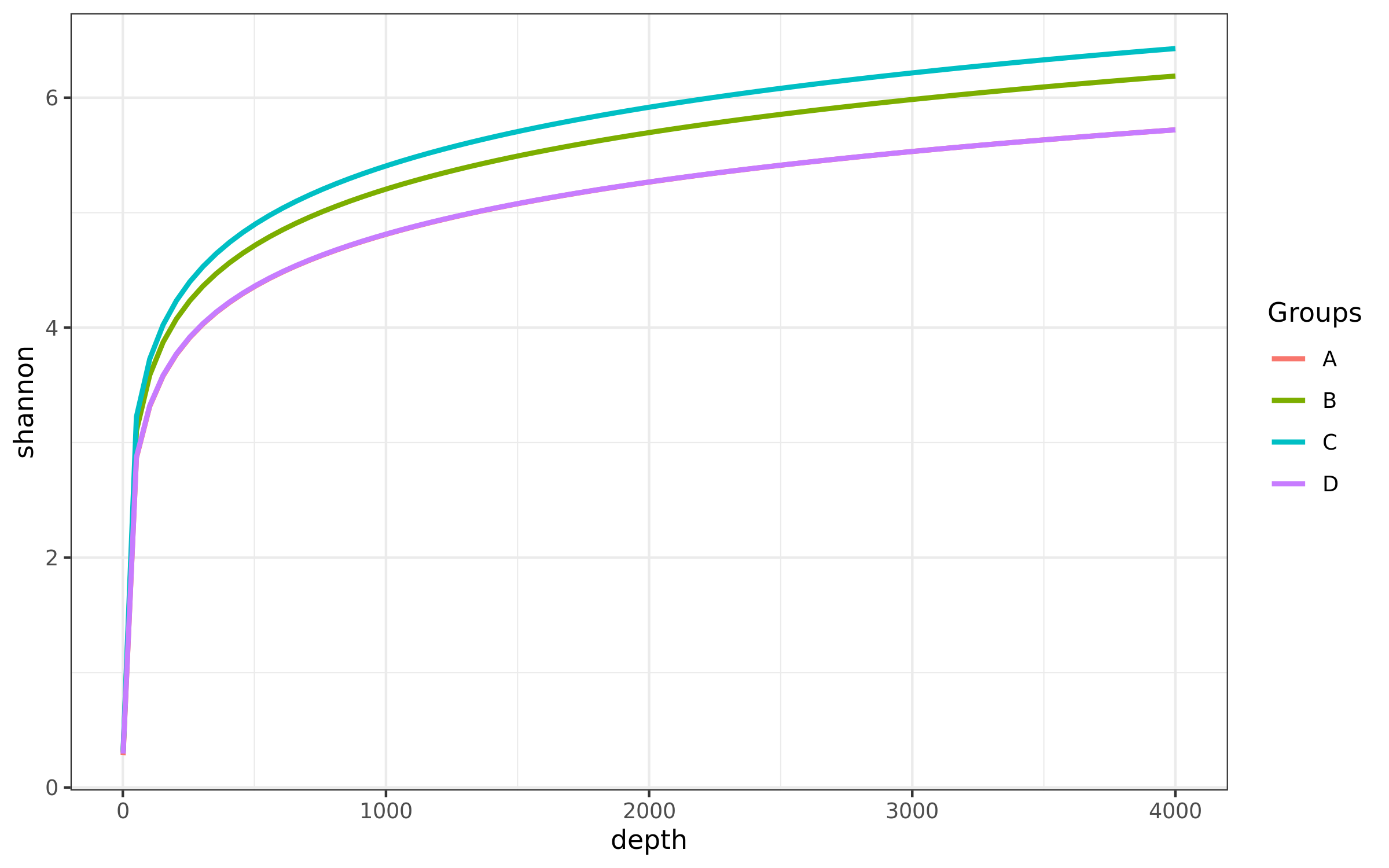

Supplement: Supplementary file 1 [file biology-14-00715-s001.zip › Supplementary Materials S1: 16s-report of gut microbiota/03_diversity-metrics/alpha_rarefaction/shannon_groups.png]

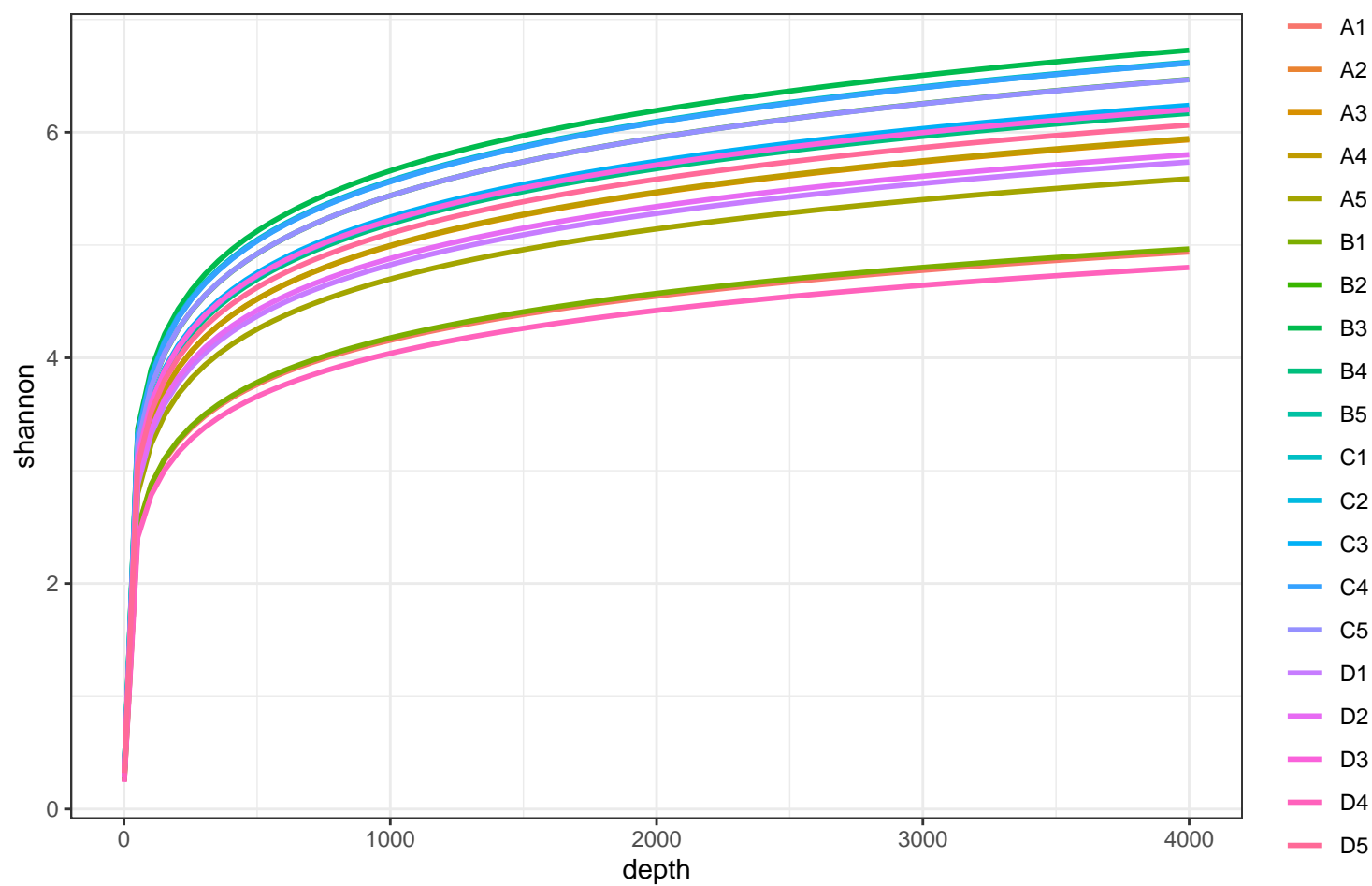

Supplement: Supplementary file 1 [file biology-14-00715-s001.zip › Supplementary Materials S1: 16s-report of gut microbiota/03_diversity-metrics/alpha_rarefaction/shannon_samples.pdf]

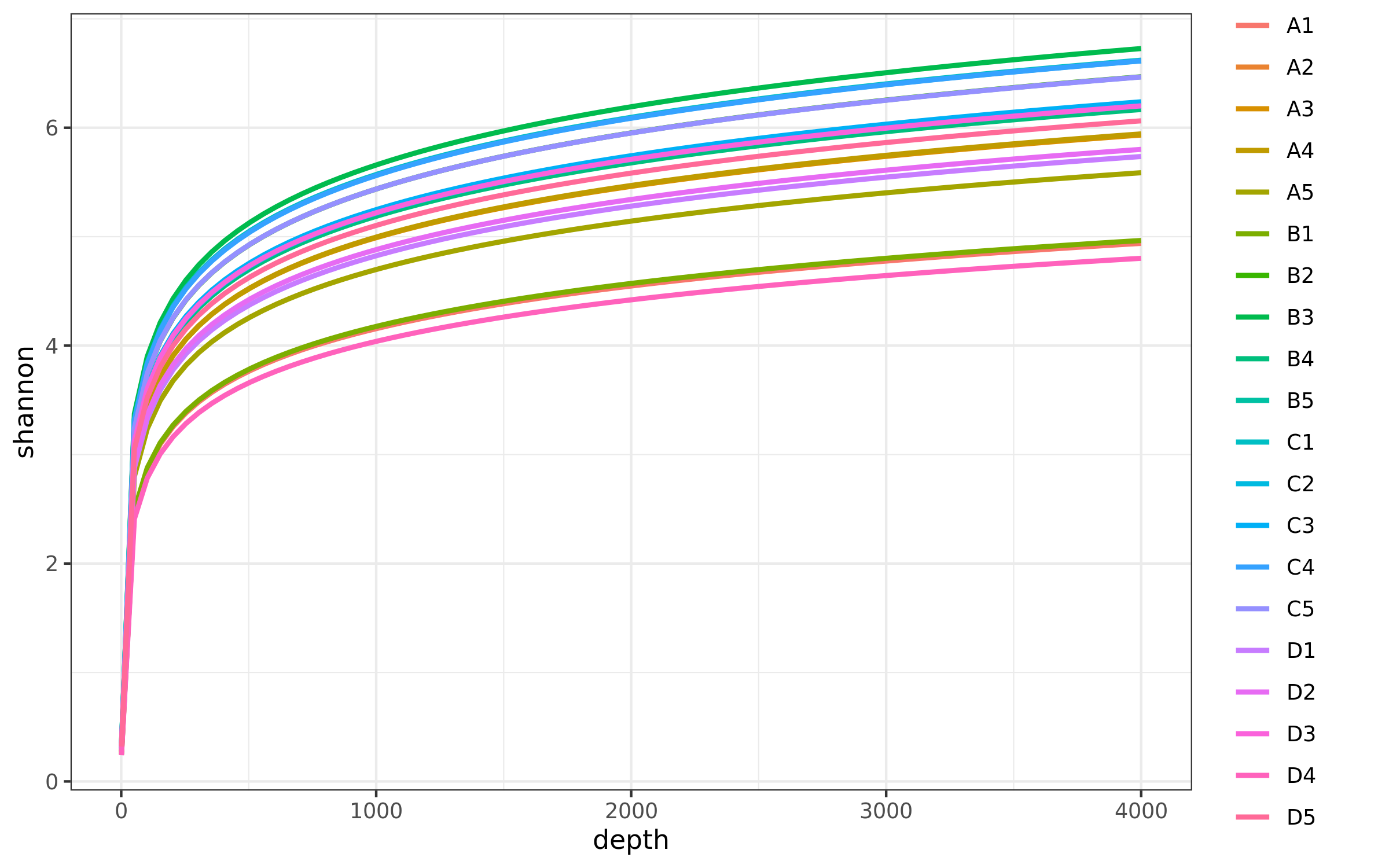

Supplement: Supplementary file 1 [file biology-14-00715-s001.zip › Supplementary Materials S1: 16s-report of gut microbiota/03_diversity-metrics/alpha_rarefaction/shannon_samples.png]

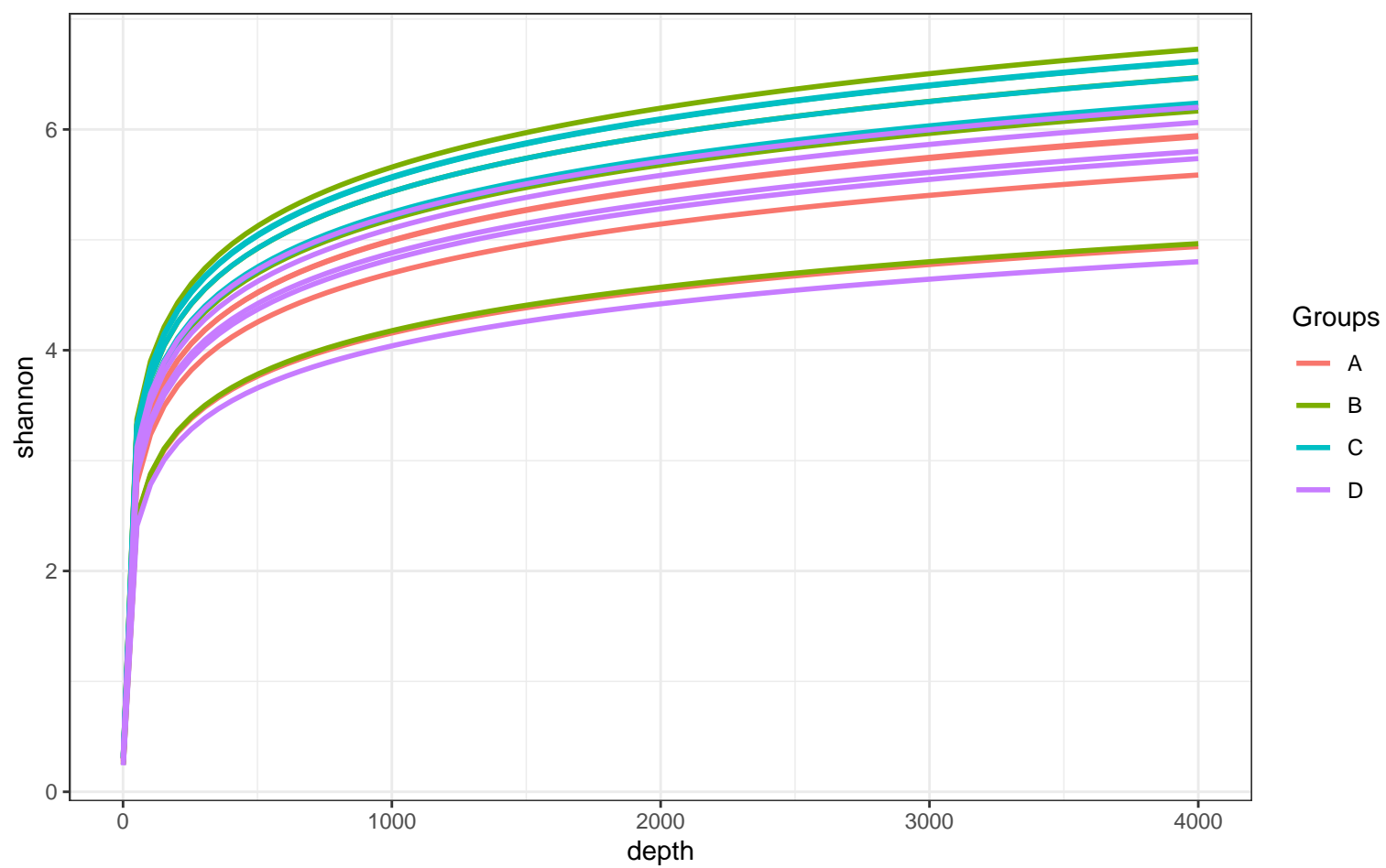

Supplement: Supplementary file 1 [file biology-14-00715-s001.zip › Supplementary Materials S1: 16s-report of gut microbiota/03_diversity-metrics/alpha_rarefaction/shannon_sample_GroupColor.pdf]

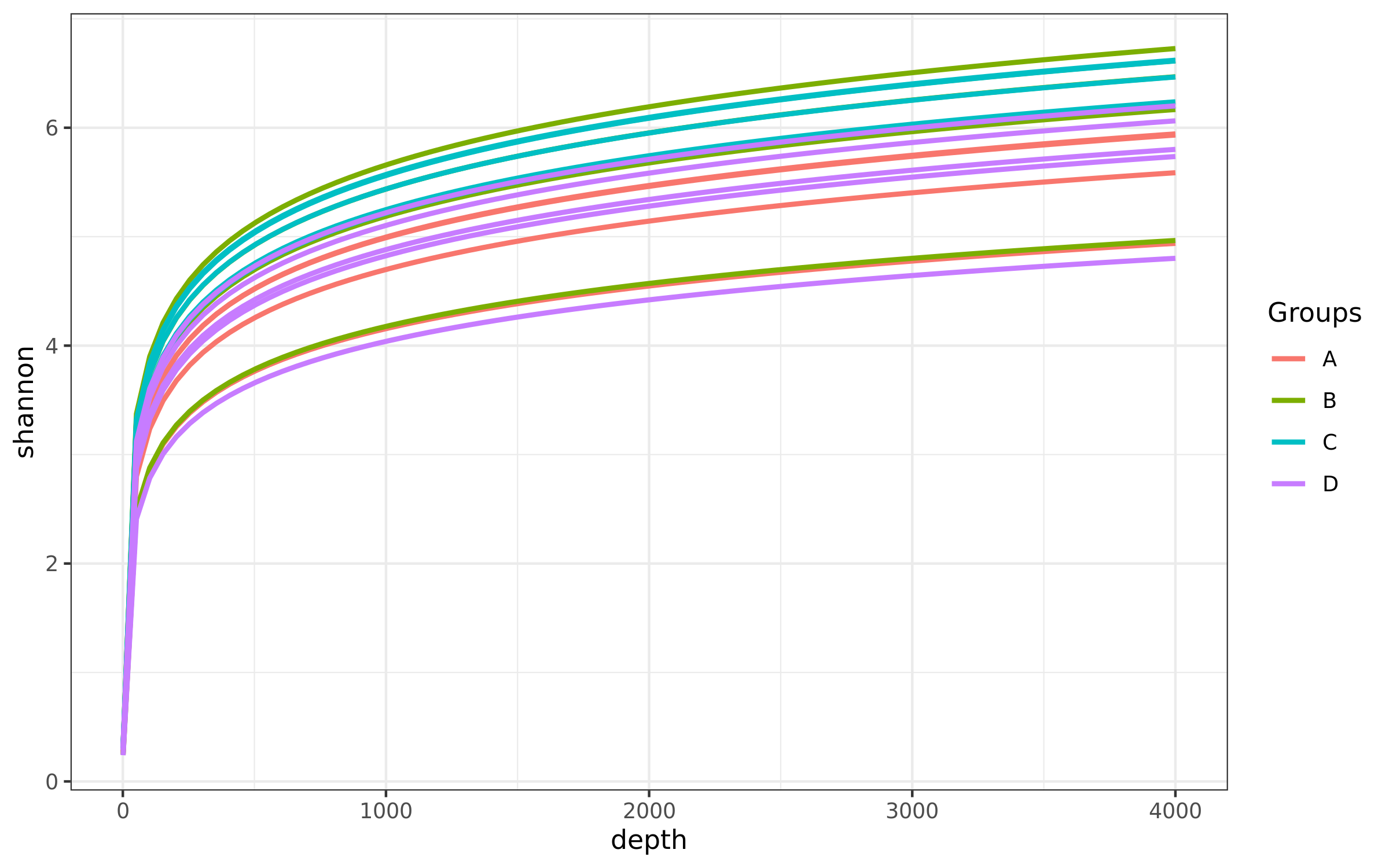

Supplement: Supplementary file 1 [file biology-14-00715-s001.zip › Supplementary Materials S1: 16s-report of gut microbiota/03_diversity-metrics/alpha_rarefaction/shannon_sample_GroupColor.png]

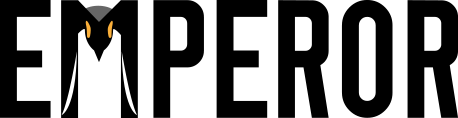

Supplement: Supplementary file 1 [file biology-14-00715-s001.zip › Supplementary Materials S1: 16s-report of gut microbiota/03_diversity-metrics/bray_curtis_emperor/img/emperor.png]

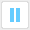

Supplement: Supplementary file 1 [file biology-14-00715-s001.zip › Supplementary Materials S1: 16s-report of gut microbiota/03_diversity-metrics/bray_curtis_emperor/img/pause.png]

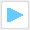

Supplement: Supplementary file 1 [file biology-14-00715-s001.zip › Supplementary Materials S1: 16s-report of gut microbiota/03_diversity-metrics/bray_curtis_emperor/img/play.png]

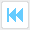

Supplement: Supplementary file 1 [file biology-14-00715-s001.zip › Supplementary Materials S1: 16s-report of gut microbiota/03_diversity-metrics/bray_curtis_emperor/img/reset.png]

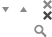

Supplement: Supplementary file 1 [file biology-14-00715-s001.zip › Supplementary Materials S1: 16s-report of gut microbiota/03_diversity-metrics/bray_curtis_emperor/vendor/css/chosen-sprite.png]

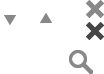

Supplement: Supplementary file 1 [file biology-14-00715-s001.zip › Supplementary Materials S1: 16s-report of gut microbiota/03_diversity-metrics/bray_curtis_emperor/vendor/css/chosen-sprite@2x.png]

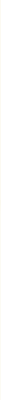

Supplement: Supplementary file 1 [file biology-14-00715-s001.zip › Supplementary Materials S1: 16s-report of gut microbiota/03_diversity-metrics/bray_curtis_emperor/vendor/css/images/ui-bg_glass_55_fbf9ee_1x400.png]

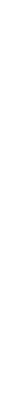

Supplement: Supplementary file 1 [file biology-14-00715-s001.zip › Supplementary Materials S1: 16s-report of gut microbiota/03_diversity-metrics/bray_curtis_emperor/vendor/css/images/ui-bg_glass_65_ffffff_1x400.png]

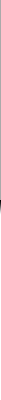

Supplement: Supplementary file 1 [file biology-14-00715-s001.zip › Supplementary Materials S1: 16s-report of gut microbiota/03_diversity-metrics/bray_curtis_emperor/vendor/css/images/ui-bg_glass_75_dadada_1x400.png]

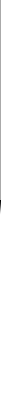

Supplement: Supplementary file 1 [file biology-14-00715-s001.zip › Supplementary Materials S1: 16s-report of gut microbiota/03_diversity-metrics/bray_curtis_emperor/vendor/css/images/ui-bg_glass_75_e6e6e6_1x400.png]

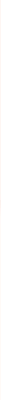

Supplement: Supplementary file 1 [file biology-14-00715-s001.zip › Supplementary Materials S1: 16s-report of gut microbiota/03_diversity-metrics/bray_curtis_emperor/vendor/css/images/ui-bg_glass_95_fef1ec_1x400.png]

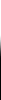

Supplement: Supplementary file 1 [file biology-14-00715-s001.zip › Supplementary Materials S1: 16s-report of gut microbiota/03_diversity-metrics/bray_curtis_emperor/vendor/css/images/ui-bg_highlight-soft_75_cccccc_1x100.png]

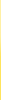

Supplement: Supplementary file 1 [file biology-14-00715-s001.zip › Supplementary Materials S1: 16s-report of gut microbiota/03_diversity-metrics/bray_curtis_emperor/vendor/css/images/ui-bg_highlight-soft_75_ffe45c_1x100.png]

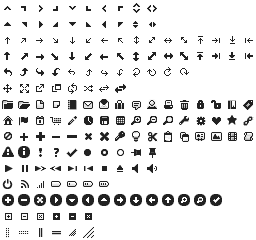

Supplement: Supplementary file 1 [file biology-14-00715-s001.zip › Supplementary Materials S1: 16s-report of gut microbiota/03_diversity-metrics/bray_curtis_emperor/vendor/css/images/ui-icons_222222_256x240.png]

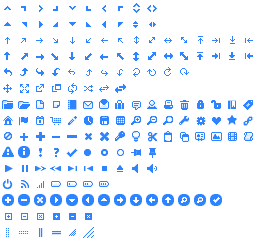

Supplement: Supplementary file 1 [file biology-14-00715-s001.zip › Supplementary Materials S1: 16s-report of gut microbiota/03_diversity-metrics/bray_curtis_emperor/vendor/css/images/ui-icons_2e83ff_256x240.png]

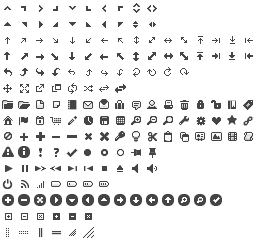

Supplement: Supplementary file 1 [file biology-14-00715-s001.zip › Supplementary Materials S1: 16s-report of gut microbiota/03_diversity-metrics/bray_curtis_emperor/vendor/css/images/ui-icons_454545_256x240.png]

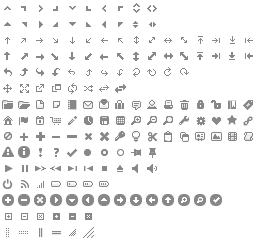

Supplement: Supplementary file 1 [file biology-14-00715-s001.zip › Supplementary Materials S1: 16s-report of gut microbiota/03_diversity-metrics/bray_curtis_emperor/vendor/css/images/ui-icons_888888_256x240.png]

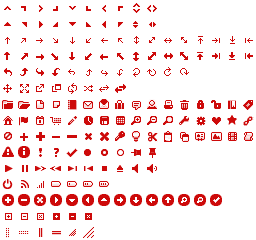

Supplement: Supplementary file 1 [file biology-14-00715-s001.zip › Supplementary Materials S1: 16s-report of gut microbiota/03_diversity-metrics/bray_curtis_emperor/vendor/css/images/ui-icons_cd0a0a_256x240.png]
